# Transcriptome Analyses Reveal Essential Roles of Alternative Splicing Regulation in Heat-Stressed Holstein Cows

**DOI:** 10.3390/ijms231810664

**Published:** 2022-09-14

**Authors:** Lirong Hu, Abdul Sammad, Congcong Zhang, Luiz F. Brito, Qing Xu, Yachun Wang

**Affiliations:** 1 National Engineering Laboratory for Animal Breeding, Key Laboratory of Animal Genetics, Breeding and Reproduction, MARA, College of Animal Sciences and Technology, China Agricultural University, Beijing 100193, China; 2 College of Life Sciences and Bioengineering, Beijing Jiaotong University, Beijing 100044, China; 3 Department of Animal Sciences, Purdue University, West Lafayette, IN 47907, USA

**Keywords:** heat stress, molecular regulations, alternative splicing, dairy cows, RNA sequencing

## Abstract

Heat stress (HS) severely impacts the productivity and welfare of dairy cows. Investigating the biological mechanisms underlying HS response is crucial for developing effective mitigation and breeding strategies. Therefore, we evaluated the changes in milk yield, physiological indicators, blood biochemical parameters, and alternative splicing (AS) patterns of lactating Holstein cows during thermoneutral (TN, N = 19) and heat-stress (HS, N = 17) conditions. There was a significant (*p* < 0.05) decline in milk yield as physiological indicators increased after exposure to natural HS conditions. The levels of eight out of 13 biochemical parameters of HS were also significantly altered in the presence of HS (*p* < 0.05). These results demonstrate that HS negatively influences various biological processes of Holstein cows. Furthermore, we investigated AS events based on the RNA-seq data from blood samples. With HS, five common types of AS events were generally increased by 6.7–38.9%. A total of 3470 AS events corresponding to 3143 unique genes were differentially alternatively spliced (DSGs) (*p*-adjusted < 0.05) between TN and HS groups. The functional annotation results show that the majority of DSGs are involved in mRNA splicing and spliceosomal complex, followed by enrichment in immune and metabolic processes. Eighty-seven out of 645 differentially expressed genes (DEGs) (fold change ≥ 1.5 and false discovery rate < 0.05) overlapped with DSGs. Further analyses showed that 20 of these genes were significantly enriched for the RNA splicing, RNA binding, and RNA transport. Among them, two genes (*RBM25* and *LUC7L3*) had strong interrelation and co-expression pattern with other genes and were identified as candidate genes potentially associated with HS responses in dairy cows. In summary, AS plays a crucial role in changing the transcriptome diversity of heat-stress-related genes in multiple biological pathways and provides a different regulation mechanism from DEGs.

## 1. Introduction

When exposed to high temperatures and relative humidity, animals experience heat stress (HS) after failing to balance metabolic heat production and heat loss with a substantial increase in body temperature. Subsequently, HS reduces livestock productivity [1,2], reproduction performance [2], and immunity [3], as a consequence of using additional energy to counterbalance the excessive heat load. In general, dairy cows are more susceptible to HS due to the fact that large animals have small surface areas per unit of weight for heat dissipation [4]. Moreover, the intensive selection for high milk yield coupled with the large amount of metabolic heat generated during the process of milk production can further aggravate the state of HS [5]. As reviewed by Sammad et al. [6], HS negatively impacts the production, reproduction, health, and welfare of dairy cows around the around.

Breeding cattle with improved thermotolerance based on genetic and genomic approaches can be a feasible alternative for extensive or semi-intensive production systems [7,8], where it is more difficult to control the environmental conditions. In this regard, a better understanding of the molecular mechanisms regulating HS response is paramount when designing effective breeding programs. With the recent development and wide availability of high-throughput sequencing technologies, numerous biological pathways and candidate genes associated with HS response have been identified based on differentially expressed genes in the blood [9], mammary gland [10], and intestine [11] tissues in cattle. However, there is little known about the post-transcriptional regulation in dairy cows after exposure to HS conditions, especially studies focusing on RNA splicing.

Alternative splicing (AS) is a phenomenon involving the generation of multiple transcripts from the same gene, which enhances the translation of mRNA isoforms and proteome diversity [12,13]. This process is performed by the molecular complex known as spliceosome, which can recognize splice sites in the pre-mRNA [14]. More than one transcript can be generated by recognizing different splice sites. In humans, nearly 95% of the total multi-exon genes are involved in AS [15]. As a prevalent and tightly regulated RNA processing, AS plays a crucial role in response to environmental stresses. For instance, this mechanism contributes to the response to HS and rapidly adjusts the abundance and function of stress-response components in plants [16]. An early study related to HS-induced AS on mouse cells clarified the alternatively spliced mRNA of the *HSP47* gene (heat shock protein 47) being involved in heat shock treatment [17]. Under HS conditions, AS regulates the transcriptional activity of *Drosophila HSF1* (heat shock transcription factor 1) by changing the ratio of three isoforms [18]. Recently, additional studies using RNA sequencing datasets were conducted to characterize the overall changes of HS-induced AS in rice [19], fish [20], and rats [21]. To our best knowledge, genome-wide regulations of AS have not yet been studied in dairy cows under HS conditions.

In this study, we firstly compared the compressive changes in milk yield, physiological indicators, and blood biochemical parameters between dairy cows exposed to thermoneutral and heat-stress conditions to investigate the negative impact of HS on their biological processes. Subsequently, we examined the changes in AS events and their potential functions in response to HS. The integration of the results obtained with findings in differentially expressed genes in blood enabled the identification of key biological processes and candidate genes potentially involved in dairy cows’ HS response. The results obtained provide a better understanding of post-transcriptional regulations in heat-stressed dairy cows and could be used as the basis for future breeding strategies.

## 2. Results

### 2.1. Heat Stress Affects the Productive Performance and Physiological Indicators of Holstein Cows

The average temperature-humidity index (THI) under the thermoneutral condition (TN) was 59.23 ± 4.32 which is considered to be a comfortable state, while the average THI value during the heat-stress conditions (HS) was 81.10 ± 7.32, which exceeded the threshold of 68 [22] and was considered as moderate HS. The response characteristics of lactational performance and physiological indicators of studied subjects under HS are summarized in Table 1. Both seven-day average milk yield (7AMY, −3.65 kg/day) and milk yield on the blood sampling day (MY, −5.31 kg) were significantly decreased during the HS condition when compared to animals under TN (*p* < 0.05). Furthermore, the TN and HS groups had significant differences in rectal temperature (RT), respiration rate (RR), and drooling score (DS) at all time points (0700, 1400, and 2100 h) (*p* < 0.05), with exception of no difference in RT at the 0700 h being observed (*p* > 0.05). In detail, HS cows showed a statistically significantly increased RT at 1400 h (0.32 °C) and 2100 h (0.48 °C) in comparison to TN cows. For RR, HS cows had drastically higher RR of 44.48, 63.48, and 50.15 breaths per minute at the three measurement points, respectively, than that of TN cows. Similar changing patterns were investigated for DS with differences of 0.24, 0.82, and 0.42 scores between the two groups. These results indicate that the experimental cows experienced HS and responded to HS by reducing milk yield and increasing RT, RR, and DS.

### 2.2. Heat Stress Induces Changes in Blood Biochemical Parameters of Holstein Cows

**Figure 1:**
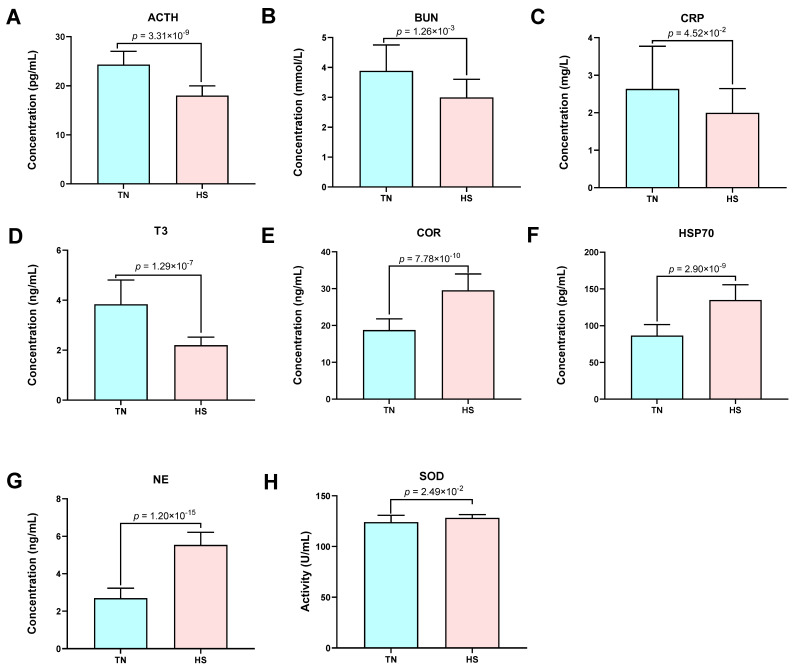
The comparisons of biochemical parameters of Holstein cows between thermal neutrality (TN) and heat stress (HS) conditions. (**A**) The comparison of adrenocorticotropic hormone (ACTH) between two groups. (**B**) The comparison of blood urea nitrogen (BUN) between two groups. (**C**) The comparison of C-reactive protein (CRP) between two groups. (**D**) The comparison of triiodothyronine (T3) between two groups. (**E**) The comparison of cortisol (COR) between two groups. (**F**) The comparison of heat shock protein 70 (HSP70) between two groups. (**G**) The comparison of norepinephrine (NE) between two groups. (**H**) The comparison of superoxide dismutase (SOD) between two groups. Data are represented as means ± standard deviation. *p* < 0.05 is considered to indicate a statistically significant difference.

Plasma samples were used to determine 13 biochemical parameters. Among them, growth hormone (GH, 3.10 ± 0.71 ng/mL versus 3.33 ± 0.44 ng/mL), glucose (GLU, 3.64 ± 0.46 mmol/L versus 3.53 ± 0.26 mmol/L), lactate (LD, 2.51 ± 0.46 mmol/L versus 2.70 ± 0.53 mmol/L), lipid peroxide (LPO, 15.15 ± 4.34 ng/mL versus 13.97 ± 1.81 ng/mL), and prolactin (PRL, 227.19 ± 20.62 uIU/mL versus 242.97 ± 54.00 uIU/mL) were not significantly different between the TN and HS groups (*p* > 0.05). The concentrations of adrenocorticotropic hormone (ACTH, −6.28 pg/mL), blood urea nitrogen (BUN, −0.88 mmol/L), C-reactive protein (CRP, −0.63 mg/L), and triiodothyronine (T3, −1.64 ng/mL) were significantly lower when cows were exposed to HS (*p* < 0.05; Figure 1A–D). Their concentrations in TN were 24.30 ± 2.71 pg/mL, 3.88 ± 0.87 mmol/L, 2.63 ± 1.14 mg/L, and 3.84 ± 0.97 ng/mL, respectively, and displayed 18.02 ± 1.95 pg/mL, 3.00 ± 0.61 mmol/L, 2.00 ± 0.61 mg/L, 2.20 ± 0.32 ng/mL in HS conditions. By contrast, cortisol (COR, 10.81 ng/mL), heat shock protein 70 (HSP70, 48.39 pg/mL), norepinephrine (NE, 2.84 ng/mL), and superoxide dismutase (SOD, 4.20 U/mL) had significantly higher levels in animals exposed to HS conditions (*p* < 0.05; Figure 1E–H). The observed values were 18.74 ± 3.05 ng/mL versus 29.56 ± 4.43 ng/mL for COR, 86.62 ± 14.98 pg/mL versus 135.01 ± 20.69 pg/mL for HSP70, 2.70 ± 0.53 ng/mL versus 5.54 ± 0.67 ng/mL for NE, and 124.03 ± 6.71 U/mL versus 128.23 ± 3.24 U/mL for SOD.

### 2.3. Descriptive Statistics of the RNA Sequencing Data

On average, 56.43 million raw reads per sample with 97.36% of Q20 and 93.47% of Q30 were obtained. An average of 53.90 million clean reads were available for further mapping after removing poor quality reads (including very short reads, low complexity reads, and low-quality reads), and the percentages of Q20 and Q30 reached 98.08% and 94.37%, respectively. Out of these, 91.82% of high-quality reads were successfully mapped to the bovine reference genome and 88.41% of reads were identified to be uniquely mapped. All samples had unique mapping rates of over 80%. These results indicate the high quality of the RNA sequencing data obtained. A statistical summary of RNA sequencing data for each sample is shown in Table S1.

### 2.4. Overview of Alternative Splicing Events

**Figure 2:**
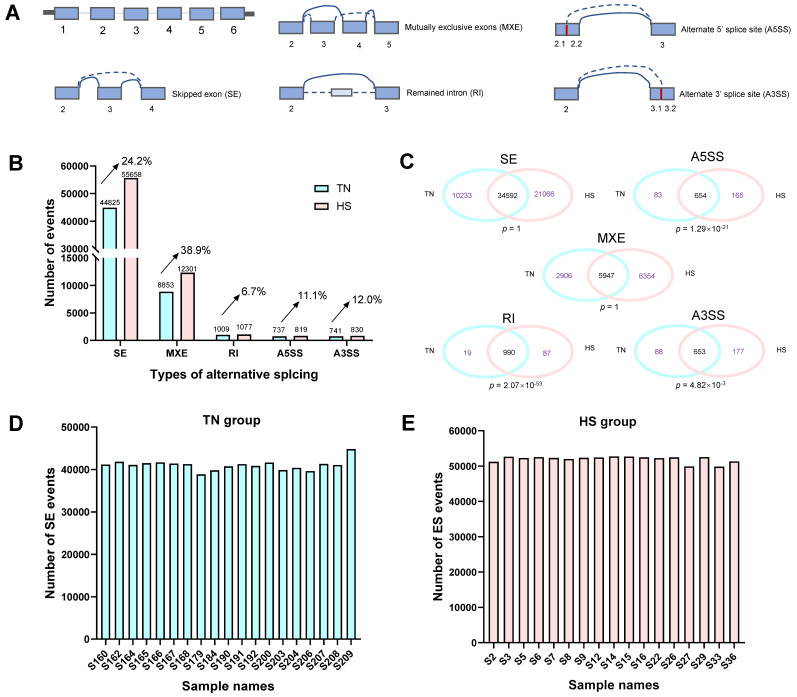
Alternative splicing (AS) events were identified at the group level and the sample levels. (**A**) Five determined AS types: skipped exon (SE), mutually exclusive exons (MXE), remained intron (RI), alternate 5′ splice site (A5SS), and alternate 3′ splice site (A3SS). (**B**) Total number of AS events categorized based on AS types in thermal neutrality (TN) and heat stress (HS) conditions. (**C**) Comparisons of the five AS types between the TN and HS groups. (**D**) Number of SE events at the sample level for the TN group. (**E**) Number of SE events at the sample level for the HS group.

**Figure 3:**
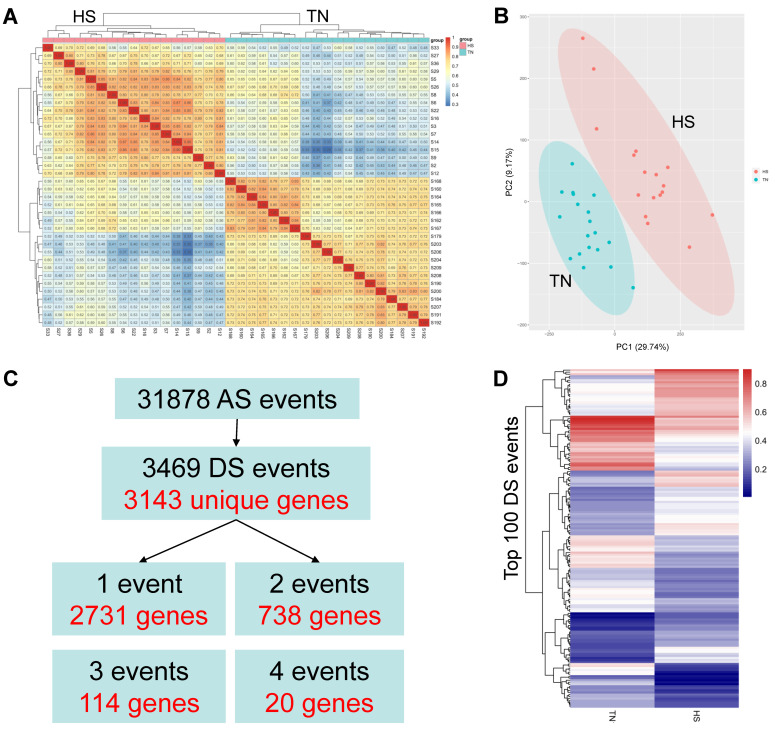
Alternative splicing (AS) profiles and differential splicing (DS) events between the thermal neutrality (TN) and heat stress (HS) groups. (**A**) Pearson correlation analysis of alternative splicing profiles among all samples. (**B**) Principal component analysis of alternative splicing profiles of TN and HS groups. (**C**) Differential splicing events between TN and HS groups. (**D**) Heatmap of the top 100 DS events, colored by percent spliced in (PSI) values.

Five types of AS events (Figure 2A), including skipped exon (SE), mutually exclusive exons (MXE), remained intron (RI), alternate 5′ splice site (A5SS), and alternate 3′ splice site (A3SS) were identified using the rMATS software. After HS, all types of AS events were increased, in which HS induced 38.9% increases in MXE and 24.4% increases in SE (Figure 2B). In terms of the comparisons of AS events between the TN and HS groups, we found that the RI, A5SS, and A3SS events had significant overlapping (*p* < 0.05), while overlapping elements were not significant for MXE and SE events (Figure 2C) (*p* > 0.05). Figure 2D,E show the number of SE events that occurred in each sample. The difference between the TN and HS groups was statistically significantly significant (*p* < 0.05). Furthermore, correlation analysis among all samples and principal component analysis (PCA) were used to investigate AS profile of the two groups based on the usage of introns (LeafCutter software), as shown in Figure 3A,B. The identified events showed a higher correlation among samples from the same group with average values of 73.50% for TN and 76.15% for HS, while an average value of 52.35% was observed among samples from different groups. Moreover, an obvious separation was observed between TN and HS in the PCA score plot of the first two principal components (Figure 2B, PC1 = 29.74%, PC2 = 9.17%). These results emphasize the difference between the AS profiles of the two groups.

### 2.5. Alternative Splicing Events Differentially Expressed between the Two Groups

In total, 31,878 events corresponding to 9701 unique genes were used to perform the differential analysis. A total of 3469 differentially alternatively spliced (DS) events corresponding to 3143 unique genes (DSGs) were identified according to the criteria *p*-adjusted value < 0.05, among which 2731, 738, 114, and 20 genes annotated contained one, two, three, and four DS events, respectively (Figure 3C, Table S2). The expression profile of the top 90 DS events is illustrated in Figure 3D, showing an expected difference between TN and HS groups. Additionally, the top 10 DS events annotated 11 genes, including *TYROBP*, *AGTRAP*, *MAP3K8*, ENSBTAG00000053845, *BCAP31*, *JPT1*, *NT5C*, *CCDC107*, *ILK*, *HNRNPF*, and *VAMP5* (Table 2). These genes are mainly involved in the immune signaling pathways. Interestingly, all of the 11 genes had a lower percent spliced in (PSI, a score to evaluate the level of specific AS events) for the HS group.

### 2.6. Functional Annotation of Genes with Differentially Alternatively Spliced Events

**Figure 4:**
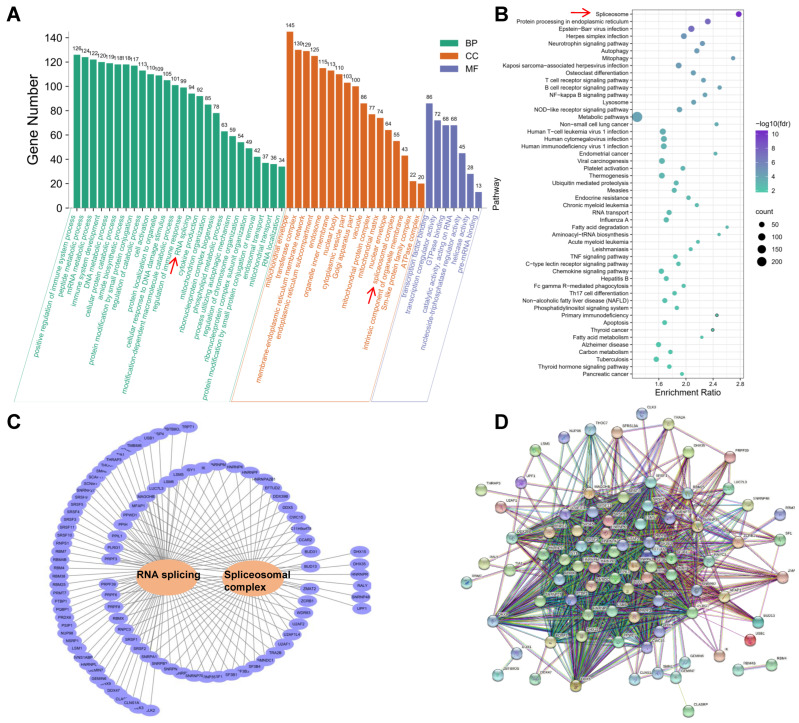
Gene Ontology (GO) and Kyoto Encyclopedia of Genes and Genomes (KEGG) annotation for differentially spliced genes. (**A**) GO terms. (**B**) KEGG pathways. (**C**) Interaction networks of genes involved in RNA splicing and spliceosomal complex. (**D**) Protein-protein interaction (PPI) of genes involved in RNA splicing and spliceosomal complex. BP: biological processes; CC: cellular components; MF: molecular functions.

The GO and KEGG analyses were carried out for functional annotation using 3143 DSGs. A total of 97 GO terms were significantly enriched (FDR < 0.05, Table S3), of which 58 biological processes (BP), 23 cellular components (CC), and 16 molecular functions (MF) were identified. The top 50 GO terms are shown in Figure 4A, including terms related to the immune system (including positive regulation of immune system process, immune system development, regulation of immune response, cytokine production, and cell activation), energy metabolic process (including mitochondrial envelope, mitochondrial protein complex, modification-dependent macromolecule catabolic process, mitochondrial matrix, peptide metabolic process, cellular protein catabolic process, phospholipid metabolic process, and regulation of catabolic process), gene expression regulation (including RNA splicing, mRNA metabolic process, spliceosomal complex, pre-mRNA binding, helicase activity, regulation of chromosome organization, transcription coregulator activity, catalytic activity acting on RNA, transcription factor binding, RNA localization, and DNA metabolic process), and cellular transportation (including endosomal transport and mitochondrial transport) were identified. Furthermore, 82 KEGG pathways are presented in Table S4. Figure 4B displays the top 50 pathways, including spliceosome, protein processing in the endoplasmic reticulum, neurotrophin signaling pathway, autophagy, apoptosis, and immune signaling pathway (including T cell receptor signaling pathway, B cell receptor signaling pathway, NF-kappa B signaling pathway, Th17 cell differentiation, chemokine signaling pathway, TNF signaling pathway, and C-type lectin receptor signaling pathway), and energy metabolism (including mitophagy, metabolic pathways, thermogenesis, fatty acid degradation, fatty acid metabolism, carbon metabolism, and thyroid hormone signaling pathway). Of these, 94 and 55 genes were significantly enriched in the process of RNA splicing (FDR = 1.11 × 10^−16^, Table S5) and spliceosomal complex (FDR = 1.90 × 10^−11^, Table S6), respectively, suggesting that DSGs also regulate the functions of splicing factors themselves. Figure 4C,D show that the genes involved in the splicing process are highly similar and interactive.

### 2.7. Differentially Alternatively Spliced Events in Differentially Expressed Genes

**Figure 5:**
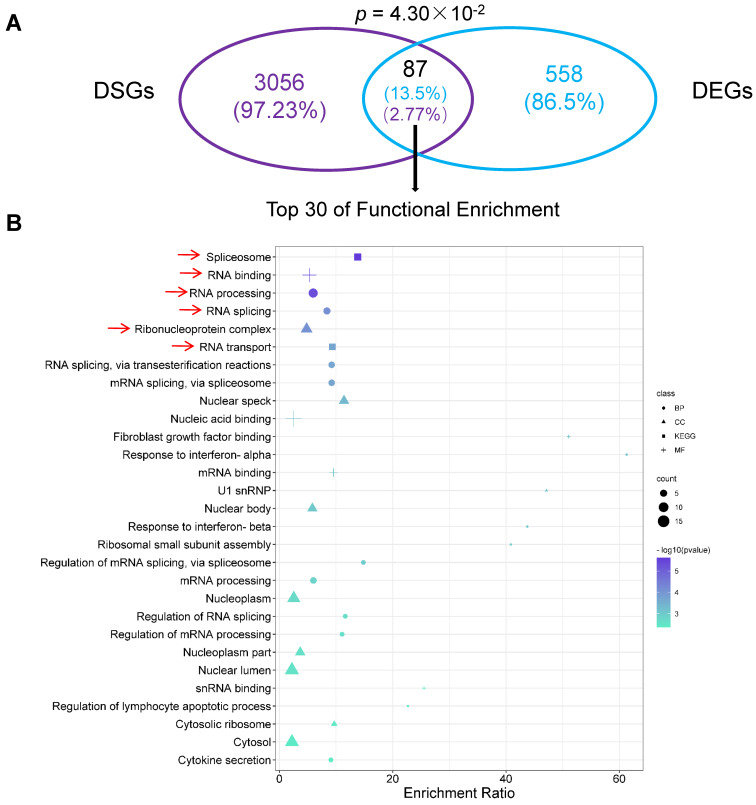
Genes presented in both differentially spliced (DSGs) and differentially expressed (DEGs). (**A**) Venn diagram of DSGs and DEGs. (**B**) Functional annotation of the overlapping genes. BP: biological processes; CC: cellular components; MF: molecular functions.

**Figure 6:**
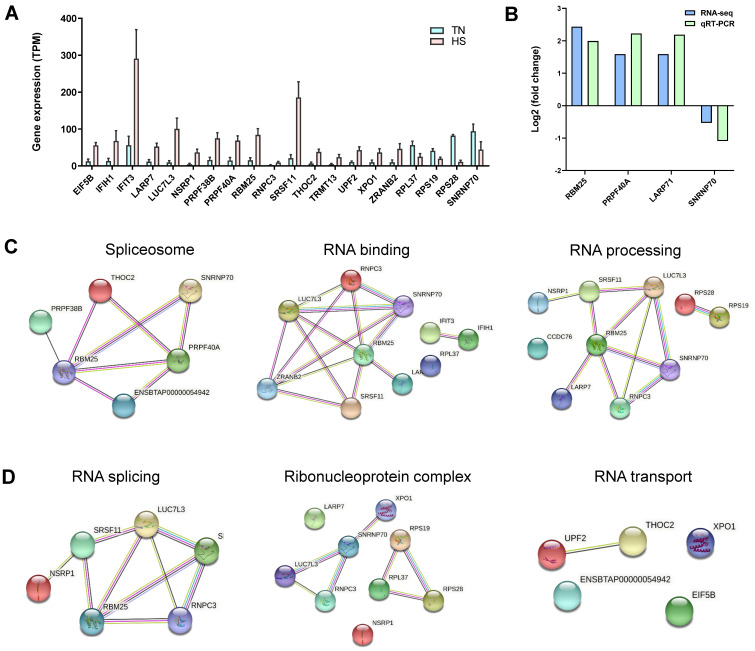
The expression patterns and protein-protein interactions of 20 genes involved in heat stress regulation. (**A**) Expression levels of 20 genes generated from RNA-seq. The values for *RPL37*, *RPS19*, and *RPS28* present as 100 times smaller. TPM: transcripts per million. (**B**) Comparative analysis of the expression levels of five selected genes between RNA-seq and real-time quantitative PCR (qRT-PCR). (**C**) Protein-protein interaction of genes involved in the spliceosome, RNA binding, and RNA processing. (**D**) Protein-protein interaction of genes involved in RNA splicing, ribonucleoprotein complex, and RNA transport.

In total, 645 differentially expressed genes (DEGs) were identified in the comparison of TN versus HS, in which 400 genes were upregulated and 245 genes were downregulated (Table S7). Further, 87 genes were determined to be DS genes corresponding to 13.5% in DEGs while 2.77% in DSGs (Figure 5A, Table S8). Functional annotation showed that they were most significantly enriched in the processes of gene expression regulation, such as spliceosome, RNA binding, RNA processing, RNA splicing, ribonucleoprotein complex, and RNA transport (Figure 5B). In total, 20 corresponding genes were identified, in which 16 genes were found to be upregulated under HS and four genes were downregulated (Figure 6A). The log-transformed relative expression fold change of four selected genes in the TN and HS groups generated from RT-qPCR were in line with the results of RNA-seq data (Figure 6B). Further PPI network analysis demonstrated that genes involved in the first four functional enrichments were strongly related and co-expressed with each other (Figure 6C). Among them, *RBM25* and *LUC7L3* can be selected as hub genes.

### 2.8. Gene Analysis for Differentially Alternative Spliced Events

**Figure 7:**
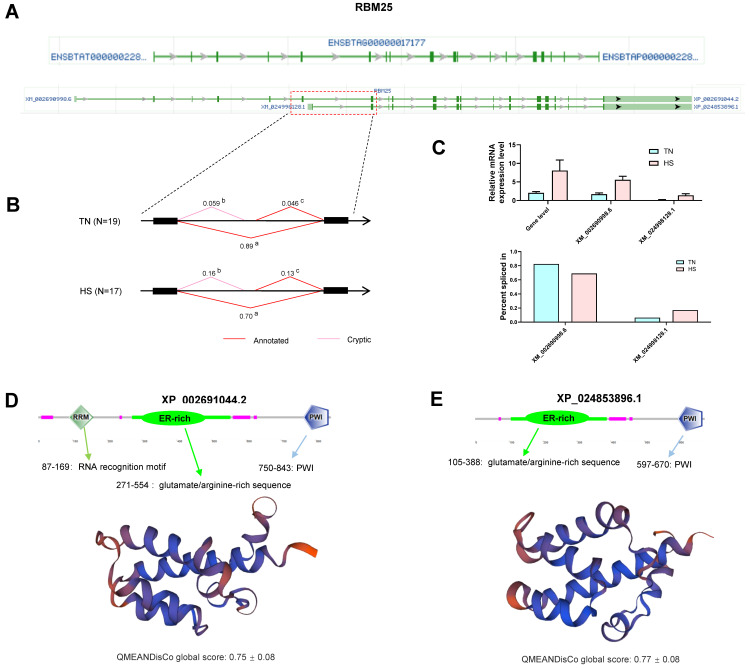
Alternative splicing events in the RBM25 gene. (**A**) Genomic regions and annotated transcript isoforms of the RBM25 gene. (**B**) Differentially alternative spliced events between thermal neutrality (TN) and heat stress (HS) groups. (**C**) Expression verification of identified events through real-time quantitative PCR (qRT-PCR). (**D**) Functional domains and three-dimensional structures of XP_002691044.2. (**E**) Functional domains and three-dimensional structures of XP_024853896.1. The QMEANDisCo global score is the average per-residue score estimated from the models.

The representative gene *RBM25* (ENSBTAG00000017177) is one of the hub genes that were significantly alternatively spliced and differentially expressed under elevated temperature. Its annotated splicing events will generate two transcript isoforms in cattle (Figure 7A), namely isoform X1 (XM_002690998.6) and isoform X2 (XM_024998128.1). In this study, as shown in Figure 7B, besides two annotated isoforms (“a” is the isoform XM_002690998.6 and “c” is the isoform XM_024998128.1), one more cryptic isoform “b” was found. At the group level, HS induced a lower proportion of the “a” isoform (70% in HS and 89% in TN) and higher proportions of the “c” isoform (13% in HS and 4.8% in TN) and “b” isoform (16% in HS and 5.9% in TN). The results of qRT-PCR also verified these detected changes (Figure 7C). We further investigated the number of amino acids and functional domains of proteins regarding to two annotated transcripts. The residue lengths of two functional proteins (XP_002691044.2 and XP_024853896.1) are 843 and 677, respectively, and domains of RNA recognition motif (RRM), glutamate/arginine-rich sequence (ER-rich), and PWI present in XP_002691044.2 while ER-rich and PWI appear in XP_024853896.1 (Figure 7D,F). Three-dimensional structure models also show significant differences between the two proteins.

## 3. Discussion

### 3.1. Effects of Heat Stress on Physiological Indicators and Milk Production

HS negatively affects the health, welfare, production, and reproduction of dairy cows. When failing to lose excessive body heat, animals tend to suffer from HS [23]. Heat-stressed cows activate a variety of physiological, biochemical, endocrine, and behavioral mechanisms to counteract HS. Body temperature measurement is important in the evaluation of the stress state of dairy cows [24] and a widely accepted indicator of body temperature and thus HS [25,26]. In addition, respiration prevents hyperthermia under high ambient temperatures through the evaporation of moisture from the respiratory tract [27]. Increased respiration rate can also lead to a disturbance in body acid-base balance [6]. Thus, excessive drooling usually accompanies an increased respiration rate [28,29]. In the present study, all three indicators had statistically higher values in the HS group, suggesting the involvement of physiological thermoregulation. Milk production was significantly lower in the HS group of cows. Milk production is an energy-intensive process causing high metabolic heat production [6], which can aggravate the HS. On the other hand, the reduction in feed intake is closely related to milk production in cows subjected to chronic HS [30]. The main purpose of intake adjustment is to reduce the production of endogenous heat [31]. In light of the above discussion, it can be concluded that physiological and productive parameters indicate the presence of HS in the dairy cows included in this experiment.

### 3.2. Effects of Heat Stress on Blood Biochemical Parameters

The concentrations of COR and NE significantly increased while ACTH and T3 decreased in HS cows in comparison to the TN cows. In terms of ACTH and COR, they are typical stress response regulators in the hypothalamic–pituitary–adrenal (HPA) axis, COR will increase after HS exposure for a long duration [32], while decreased ACTH is related to the feedback mechanisms [33]. Moreover, the sympathetic-adrenal-medullary (SAM) axis coordinates the responses of HS by mediating the release of NE, which helps to regulate the cardiopulmonary system, such as by increasing the RR [34]. Thyroid hormones (e.g., T3) and its hypothalamic-pituitary-thyroid (HPT) axis are the components of the metabolic pathway in regulating metabolic heat production. Their lower activities cause a reduction of metabolic energy when animals are coping with high-temperature conditions [35]. Under HS conditions, other functional molecules also play an important role in response to HS. For instance, HS causes oxidative stress that stimulates certain body defense mechanisms and increases oxidative stress-related molecules such as SOD [36]. The HSP70 is activated in the presence of HS and protects cells from damage in oxidative stress [37]. Our results show that exposure to HS conditions increased both SOD and HSP70 levels in the plasma of dairy cows, which is in agreement with previous studies [36,37]. CRP as a member of the pentraxin family is known as a sensitive inflammation marker [38]. The decrease in CRP may be due to the liver dysfunction caused by HS. Considering the above, we conclude that cows suffering from HS exposure modulate the levels of hormones and functional molecules, implicating the complex regulation of HS response in dairy cows.

### 3.3. Effects of Heat Stress on Alternative Splicing

Heat stress response is a very complex process that involves gene regulation in multiple tissues and different molecular levels. Exposure to elevated temperature causes a sequence of changes in heat shock factors and transcriptional systems, and then alters extensive gene expression in response to HS [39]. Numerous studies have been reported on the regulatory mechanisms of HS response, particularly, focusing on the gene expression patterns and those synchronously changed networks [9,40]. Moreover, heat-inducible splicing regulation of heat-responsive genes has been also reported in different species [21,41]. In the comparison with other types of stress, the interaction between AS and HS is stronger [42]. The process for RNA splicing is regulated by cis-regulatory elements in pre-mRNA and trans-regulatory elements, mainly RNA-binding proteins (RBPs) [43]. A group of RBPs gets involved in AS regulation under HS via modulation of their own transcription and post-transcription, as well as phosphorylation status [16]. Recent reports indicate that AS is a type of positive regulatory mechanism to enable organisms to better cope with HS. For instance, grapes utilized AS to maintain the resource balance under hot conditions by adjusting their physiology and metabolism [44]. Hundreds of genes in catfish showed the involvement of RNA splicing-related genes underlying heat tolerance [41]. However, the functional roles of AS regulation in heat-stressed cows are very limited.

Accordingly, we conducted a global analysis of AS induced by HS in Holstein cows. We found that the number of AS events was higher during the HS period, which is consistent with the study in fish [41]. These stress-induced increases in AS events were also reported in rats [21] and pigs [45]. A total of 3143 unique DSGs were found between the TN and HS groups. The top genes are mainly involved in immune response and cell adaptation. These genes found in this study are important, as a recent study involving RNA sequencing of TN versus HS Holstein cows showed that immune effector response was predominant [46]. Similarly, another study investigating the effect of acute heat stress on bovine granulosa cells also found the upregulation of a high proportion of inflammation-related genes [40]. A gene involving high DS events, the *ATGRAP* gene, is pivotal due to its direct relationship with angiotensin II. Angiotensin II is particularly related to vasoconstriction and aldosterone biosynthesis [47,48]. Along with glucocorticoids, aldosterone initially tends to increase for homeostatic mechanisms in response to HS [49]. Furthermore, we performed the functional annotation of all DSGs. The results illustrate that these functional genes are involved in the immune system, metabolism, gene expression, and cellular transportation. This suggests that AS plays a crucial role in changing the transcriptome diversity of heat-stress-related genes in multiple biological processes. Notably, 94 and 55 genes were significantly enriched in the process of mRNA splicing and spliceosomal complex, respectively. These results are consistent with the findings in previous studies showing that splicing factors themselves also underwent regulations by AS in response to external stresses [41,50]. Therefore, DS events could regulate the functions of splicing factors themselves.

In order to better understand the regulatory mechanisms of HS, we then focused on the overlapping genes between DSGs and DEGs because experimental cows were able to cope with HS by changing both gene expression and transcript diversity. In detail, 87 DSGs (2.77% of all DSGs) related to HS were identified with differential expression at the transcriptional level and only constituted 13.5% of the total DEGs, indicating that heat-inducible AS has an independent pattern of regulation that largely differs from transcriptional regulation [21]. Therefore, there is a need for integrating the results of gene expression, gene splicing, and various regulatory mechanisms to better understand the complex transcriptional responses to HS. Surprisingly, we also found the coordinated processes in gene expression regulation (such as RNA splicing, RNA binding, and RNA transport) based on the functional analyses for those common genes, in which 20 genes were identified. Among them, RBM25, a functional protein localizes to the nuclear speckles, showed a strong interrelation and co-expression pattern with other genes. This gene is a global and crucial splicing factor due to its survival conferring role in the cells and strong conservation across eukaryotic lineages. Moreover, RBM25 interacts with components of the early spliceosome and regulators of AS [51]. In plants, *RBM25* modulates response to drought stress by affecting the splicing process of the upstream gene *HAB1* [52]. Human *RBM25* is reported to promote apoptosis by regulating the transcripts of the gene *BCL2L1* [53]. So far, the relationship between *RBM25* and HS response is not well known. In the present study, the increased gene expression of *RBM25* was found, indicating that it is related to molecular processes and biological functions in response to HS. Notably, the proportions of its two annotated transcripts demonstrated a significant difference between the TN and HS groups. HS induced a lower proportion of XM_002690998.6 (19%) and a higher proportion of XM_024998128.1 (8.2%). The XM_002690998.6 encodes a protein with 843 amino acid lengths containing RRM, ER-rich, and PWI domains, and XM_024998128.1 encodes 677 protein residues comprising ER-rich and PWI. All three motifs as important protein domains have the structural and functional characterization of RNA binding [53]. However, a previous study reported that RRM and PWI domains could be responsible for the interactions between RBM25 and other proteins, but they were not necessary [51]. At the same time, the ER-rich was the key domain to those protein-protein interactions [51]. Therefore, the increase of XM_024998128.1 may be the result of large demands of the RBM25 protein in the response to HS as it can produce functional protein faster. Our results reveal that this gene is a critical regulator involved in the regulation of HS response. The *LUC7L3* gene, which encodes another nuclear protein with roles in pre-mRNA splicing, was also identified in this study. Its expression was found to be significantly correlated with AS events [54]. The LUC7L3 in vertebrates has two zinc finger domains, one arginine/serine-rich domain (RS), and one glutamate/arginine-rich domain (ER), in which RS is the common feature of the SR family of splicing factors [55]. LUC7L3 not only serves as a bridge between the pre-mRNA and the U1 snRNA but also interacts with *RBM25* via binding the exonic element [53]. This is consistent with our results in the PPI analysis. In our case study, the increased expression pattern of *LUC7L3* was investigated in HS, besides, heat-induced significant changes in its AS events were observed as well. This provides compelling insight into the important role of LUC7L3 in the presence of HS. Therefore, the RNA splicing-related genes were suggested to be regulated by the process of AS themselves [16], which may in turn facilitate the splicing of other pre-mRNAs. This study was performed on the basis of limited RNA-seq analysis and did not consider the indeed changes in translation level using the proteome platform. However, these results provide a reference for future research. Additional studies should be carried out to validate the reported differential AS events when cows are exposed to HS treatments.

## 4. Materials and Methods

### 4.1. Sample Collection

Animal care was undertaken in agreement with the Committee on Ethics of Animal Experimentation from the China Agricultural University (Beijing, China). A total of 36 primiparous Holstein cows at a similar lactation stage (~138 days in milk) from a commercial farm (Beijing, China) were selected as experimental animals. Experimental cows were housed in herds with an outdoor playground and fed with total mixed rations (TMR) in line with dairy feeding standard (NY/T 34-2004) and milked thrice daily (at 0700, 1400, and 2100 h). All the animals had ad libitum access to water. The temperature and humidity were recorded with an auto-recording hygrothermograph (179-THL, Apresys Inc., San Ramon, CA, USA; ± 0.3 °C and ± 3% relative humidity), and the temperature-humidity index (THI) was calculated based on the equation: THI = 0.8AT + RH × (AT-14.1) + 46.4, where AT is the ambient temperature (°C) and RH is relative humidity [56]. The experiment was conducted during two periods based on THI, which included the thermoneutral period in April (TN, N = 19) and the heat-stressed period in July (HS, N = 17) of 2017. Approximately 10 mL of blood samples were harvested from the tail vein into anticoagulant tubes with EDTA-2K and centrifuged at 1400 × *g* for 15 min. Leukocytes were collected and mixed with TRIzol (No. 15596018, Thermo, Waltham, MA, USA), then stored at −80 °C until RNA isolation. Upper plasma was used to measure the biochemical parameters.

### 4.2. Phenotypic Measurements

Average milk yield for seven continuous days (7AMY) and daily milk yield on the blood sampling day (MY) were recorded. Rectal temperature (RT) was measured using a digital thermometer with a precision of 0.1 °C (MC-347, Omron, Tokyo, Japan) in the morning (0700–0900 h), afternoon (1400–1600 h), and evening (2100–2300 h) for three continuous days. At the same time, one trained observer recorded the respiration rate (RR) of each cow with a stopwatch according to flanking movement of the cow’s body during 30 s, as well as the drooling score (DS) based on the following standard: 1, no salivation; 2, hanging or watery dripping; and, 3, drowning or inflowing [57].

### 4.3. Determination of Biochemical Parameters

The plasma concentrations of 13 biochemical parameters related to HS were determined, including adrenocorticotropic hormone (ACTH), blood urea nitrogen (BUN), cortisol (COR), C-reactive protein (CRP), growth hormone (GH), glucose (GLU), heat shock protein 70 (HSP70), lactate (LD), lipid peroxide (LPO), norepinephrine (NE), prolactin (PRL), superoxide dismutase (SOD), and triiodothyronine (T3). The method of chemical colorimetry was used to measure BUN, CRP, GLU, LD, and SOD. The radioimmunoassay (RIA) was conducted to quantify COR, GH, PRL, and T3. The enzyme-linked immunosorbent assay (ELISA) was carried out to measure ACTH, HSP70, LPO, and NE.

### 4.4. Library Preparation and RNA Sequencing

The total RNA samples were isolated from leukocytes according to the manufacturer’s instructions for TRIzol Reagent method [58]. RNA concentration and quality were determined using Equalbit RNA BR Assay Kit (Cat No. Q10211, Invitrogen, Carlsbad, CA, USA) and Nanodrop 2000 (Thermo, Massachusetts, USA). RNA integrity was assessed using 1% agarose gel electrophoresis, and it was used for library construction with 28S/18S >1. For the RNA-Seq library, 2 g total RNA was used for purification and fragment using NEBNext Poly(A) mRNA Magnetic Isolation Module (Cat No. E7490S, New England Biolabs (UK) Ltd., Hitchin, Herts, UK) then followed by cDNA library with NEBNext Ultra RNA Library Prep Kit for Illumina (Cat No. E7530S, New England Biolabs Ltd., Hitchin, Herts, UK). All libraries were quantitated based on Equalbit DNA BR Assay Kit (Invitrogen, CA, USA), pooled equimolarly, and sequenced using the NovaSeq 6000 System (Illumina Inc., San Diego, CA, USA) which generates 150 bp paired-end reads.

### 4.5. Quality Control and Reads Alignment

The FastQC software (v0.11.9) (https://www.bioinformatics.babraham.ac.uk/projects/fastqc/, accessed on 5 July 2021) was used to evaluate the quality of the sequencing reads, and then all reads were filtered with the adapter, less length, low complexity, and low quality, as well as global trimming using the Fastp software (v0.20.0) [59]. After that, clean reads were mapped to the bovine reference genome of version ARS-UCD1.2 using the STAR (v2.7.5c) software [60].

### 4.6. Differentially Expressed Gene Analyses

We investigated the counts of gene expression using the RNA-SeQC software (v2.3.6) [61]. For screening the differentially expressed genes (DEG), quantile-adjusted conditional maximum likelihood (qCML) was performed using the edgeR package in R [62] with criteria fold change (FC) ≥ 1.5 and 0.05 for the false discovery rate (FDR).

### 4.7. Alternative Splicing Analysis

The rMATs (v4.1.0) [63] and Leafcutter (v0.2.9) [64] software were used to identify AS events and significantly differential AS events based on the leverage information in bam files. Briefly, rMATs utilizes the proportions of exon-exon junction reads to calculate five AS events, including skipped exon (SE), mutually exclusive exons (MXE), remained intron (RI), alternate 5′ splice site (A5SS), and alternate 3′ splice site (A3SS). The clustering analysis of AS events in TN and HS groups was performed using the Venn2.1 package (https://bioinfogp.cnb.csic.es/tools/venny/, accessed on 31 August 2022). A hypergeometric distribution test in R software (‘phyper’ function) was performed to test whether the overlap was significant. Leafcutter obtains the excised introns from reads to infer exon-exon junctions. Given its high accuracy in AS quantification, Leafcutter was performed to determine the DS events between the two groups that satisfied the condition of *p*-adjusted < 0.05 (based on the Bonferroni adjustment, *p*-adjusted = *p*-value × the number of tests). For these novel alternative splice events, they were considered as reliable ones from which had been excised ≥ 20% of the time in ≥ 25% of the samples compared with other overlapping introns. In addition, principal component analysis (PCA), Pearson’s correlation, and heatmap analysis of the two groups (TN and HS) were performed using the R software.

### 4.8. Functional Annotation of Differentially Alternative Splicing Genes

Gene ontology (GO) enrichment and Kyoto Encyclopedia of Genes and Genomes (KEGG) pathway analyses were conducted using the online WebGestalt software (http://www.webgestalt.org/, accessed on 28 October 2021) to explore the biochemical processes and pathways that DSGs involved in. Regulators were considered to be significantly enriched based on FDR < 0.05. Protein-protein interaction (PPI) was constructed using the STRING database (https://string-db.org/, accessed on 28 October 2021).

### 4.9. Identification of Genes That Were Both Differentially Expressed and Differentially Alternatively Spliced

Overlapping analysis was conducted to cluster the differentially expressed genes and differentially alternatively spliced genes through Venn2.1 (https://bioinfogp.cnb.csic.es/tools/venny/, accessed on 29 October 2021). The significant level of overlapping genes was tested based on hypergeometric analysis using the ‘phyper’ function in the R software. Furthermore, functional enrichment analyses and interactions of these important genes were performed using WebGestalt and STRING, respectively. The effects of AS events on their annotated transcripts and protein products can be identified based on the records in the NCBI database (https://www.ncbi.nlm.nih.gov/, accessed on 29 October 2021). These corresponding changes in functional domains and three-dimensional structure were predicted using the SMART (http://smart.embl-heidelberg.de/, accessed on 1 September 2022) and SWISS-MODEL (https://swissmodel.expasy.org/interactive, accessed on 1 September 2022) websites.

### 4.10. Validation of Quantitative Real-Time PCR (qRT-PCR)

Four genes were chosen for the validation of RNA-Seq results, including *RBM25*, *PRPF40A*, *LARP71*, and *SNRNP70*, and *GAPDH* was the housekeeping gene. The Primer-BLAST tool in NCBI (https://www.ncbi.nlm.nih.gov/tools/primer-blast/, accessed on 31 August 2022) was performed to design primers based on their mRNA sequences, and the detailed description can be found in Table S9. cDNA synthesis was achieved using a PrimeScript™RT reagent Kit with gDNA Eraser (Cat No. RR047A, Takara Biotechnology (Dalian) Co., Ltd., Dalian, Liaoning, China) conducted with 500 ng of total RNA. Validation for RNA-Seq results was conducted using quantitative real-time PCR (qRT-PCR) with SYBR Green FastFire qPCR PreMix (Cat No. FP207-02, Tiangen Biotech Co., Ltd., Beijing, China). Each reaction was performed in a total volume mixture of 20 μL consisting of 1 μL cDNA as template, 0.6 μL each of primers (10 μM), 10 μL 2 × FastFire qPCR PreMix, and 7.8 μL RNase-free ddH_2_O. The amplification protocol was as follows: 95 °C for 15 min, followed by 40 cycles of 95 °C for 10 s and 60 °C for 30 s. RNA expression levels relative to the control gene were calculated as 2^−∆∆Ct^. To compare the results of the RNA-seq analysis and qRT-PCR, fold change values were log2 transformed.

## 5. Conclusions

Heat-stressed cows had a significantly higher rectal temperature, respiration rate, drooling score, and lower milk yield. In general, multiple biochemical parameters were altered during heat stress conditions in comparison to the thermoneutral period. The differentially alternatively spliced genes identified when comparing the two groups are involved in functional processes related to RNA splicing, immune system, and metabolic pathways. Moreover, low proportions of genes overlapped between differentially alternatively spliced and differentially expressed genes, highlighting their potentially independent functions in response to heat stress. Taken together, these findings indicate that alternative splicing is an important transcriptional mechanism in heat-stressed dairy cows.

## Figures and Tables

**Table 1:** Productive performance and physiological indicators of Holstein cows under thermal neutrality (TN) and heat stress (HS) conditions.

Variable	Thermal Neutrality (TN, N = 19) ^1^	Heat Stress (HS, N = 17) ^1^	*p*-Value ^2^
**Milk production**			
7AMY (kg/day)	40.60 ± 8.77	36.95 ± 4.96	3.41 × 10^−2^
MY (kg)	41.33 ± 7.67	36.02 ± 5.63	2.67 × 10^−2^
**Rectal Temperature (°C)**			
0700 h	38.53 ± 0.19	38.64 ± 0.30	1.61 × 10^−1^
1400 h	38.68 ± 0.14	39.00 ± 0.59	4.78 × 10^−2^
2100 h	38.52 ± 0.19	39.00 ± 0.63	2.68 × 10^−2^
**Respiration Rate (breaths/min)**			
0700 h	35.16 ± 3.74	79.64 ± 18.15	2.41 × 10^−8^
1400 h	40.52 ± 6.32	104.00 ± 20.30	3.06 × 10^−10^
2100 h	35.26 ± 3.74	85.41 ± 25.27	2.13 × 10^−10^
**Drooling Score**			
0700 h	1.00 ± 0.00	1.24 ± 0.43	4.13 × 10^−2^
1400 h	1.00 ± 0.00	1.82 ± 0.78	6.82 × 10^−4^
2100 h	1.00 ± 0.00	1.41 ± 0.69	2.99 × 10^−2^

Notes: 7AMY, average milk yield across seven days; MY, milk yield on the blood sampling day; RT, rectal temperature; RR, respiration rate; DS, drooling score. ^1^ Mean ± standard deviation. ^2^ *p*-values were calculated using the Student’s *t*-test.

**Table 2:** The top 10 differential alternative splicing events between Holstein cows experiencing thermal neutrality (TN) and heat stress (HS) conditions.

Event	Gene	Description	TN (PSI ^1^)	HS (PSI ^1^)	HS-TN (ΔPSI)	*p*-Adjusted ^2^
chr18:clu_38374_NA	*TYROBP*	Transmembrane immune signaling adaptor TYROBP	0.39	0.28	−0.11	1.34 × 10^−86^
chr16:clu_44038_NA	*AGTRAP*	Angiotensin II receptor associated protein	0.25	0.13	−0.12	8.68 × 10^−79^
chr13:clu_28601_NA	*MAP3K8*	Mitogen-activated protein kinase kinase kinase 8	0.35	0.04	−0.30	3.06 × 10^−78^
chr12:clu_40742_NA	ENSBTAG00000053845	Ras GTPase-activating protein 3-like	0.67	0.36	−0.32	1.17 × 10^−70^
chrX:clu_27138_NA	*BCAP31*	B cell receptor associated protein 31	0.76	0.41	−0.35	5.54 × 10^−69^
chr19:clu_25591_NA	*JPT1*; *NT5C*	Jupiter microtubule associated homolog 1; 5′,3′-nucleotidase, cytosolic	0.32	0.19	−0.13	1.54 × 10^−59^
chr8:clu_30411_NA	*CCDC107*	Coiled-coil domain containing 107	0.46	0.06	−0.40	5.80 × 10^−59^
chr15:clu_11260_NA	*ILK*	Integrin linked kinase	0.69	0.49	−0.20	4.93 × 10^−58^
chr28:clu_4496_NA	*HNRNPF*	Heterogeneous nuclear ribonucleoprotein F	0.32	0.16	−0.16	6.49 × 10^−56^
chr11:clu_41811_NA	*VAMP5*	Vesicle associated membrane protein 5	0.90	0.56	−0.35	7.50 × 10^−56^

Notes: ^1^ PSI, percent spliced in. ^2^ *p*-adjusted = *p*-value × the number of tests.

## Data Availability

All the pertinent data is presented in the manuscript and associated supplementary files. Raw sequencing data can be obtained from the corresponding author.

## Supplementary Materials

The supporting information can be downloaded at: https://www.mdpi.com/article/10.3390/ijms231810664/s1.

## References

[1] Tao S., Orellana Rivas R.M., Marins T.N., Chen Y.-C., Gao J., Bernard J.K. (2020). Impact of heat stress on lactational performance of dairy cows. Theriogenology.

[2] Menta P.R., Machado V.S., Piñeiro J.M., Thatcher W.W., Santos J.E.P., Vieira-Neto A. (2022). Heat stress during the transition period is associated with impaired production, reproduction, and survival in dairy cows. J. Dairy Sci..

[3] Dahl G.E., Tao S., Laporta J. (2020). Heat Stress Impacts Immune Status in Cows Across the Life Cycle. Front. Vet. Sci..

[4] Brody S. (1956). Climatic Physiology of Cattle1. J. Dairy Sci..

[5] Brito L.F., Bedere N., Douhard F., Oliveira H.R., Arnal M., Peñagaricano F., Schinckel A.P., Baes C.F., Miglior F. (2021). Review: Genetic selection of high-yielding dairy cattle toward sustainable farming systems in a rapidly changing world. Animal.

[6] Sammad A., Wang Y.J., Umer S., Lirong H., Khan I., Khan A., Ahmad B., Wang Y. (2020). Nutritional Physiology and Biochemistry of Dairy Cattle under the Influence of Heat Stress: Consequences and Opportunities. Animals.

[7] Ravagnolo O., Misztal I., Hoogenboom G. (2000). Genetic component of heat stress in dairy cattle, development of heat index function. J. Dairy Sci..

[8] Nguyen T.T.T., Bowman P.J., Haile-Mariam M., Pryce J.E., Hayes B.J. (2016). Genomic selection for tolerance to heat stress in Australian dairy cattle. J. Dairy Sci..

[9] Garner J.B., Chamberlain A.J., Vander Jagt C., Nguyen T.T.T., Mason B.A., Marett L.C., Leury B.J., Wales W.J., Hayes B.J. (2020). Gene expression of the heat stress response in bovine peripheral white blood cells and milk somatic cells in vivo. Sci. Rep..

[10] Yue S., Wang Z., Wang L., Peng Q., Xue B. (2020). Transcriptome Functional Analysis of Mammary Gland of Cows in Heat Stress and Thermoneutral Condition. Animals.

[11] Koch F., Thom U., Albrecht E., Weikard R., Nolte W., Kuhla B., Kuehn C. (2019). Heat stress directly impairs gut integrity and recruits distinct immune cell populations into the bovine intestine. Proc. Natl. Acad. Sci. USA.

[12] Gilbert W. (1978). Why genes in pieces?. Nature.

[13] Kelemen O., Convertini P., Zhang Z., Wen Y., Shen M., Falaleeva M., Stamm S. (2013). Function of alternative splicing. Gene.

[14] Chen W., Moore M.J. (2015). Spliceosomes. Curr. Biol..

[15] Pan Q., Shai O., Lee L.J., Frey B.J., Blencowe B.J. (2008). Deep surveying of alternative splicing complexity in the human transcriptome by high-throughput sequencing. Nat. Genet..

[16] Ling Y., Mahfouz M.M., Zhou S. (2021). Pre-mRNA alternative splicing as a modulator for heat stress response in plants. Trends Plant Sci..

[17] Takechi H., Hosokawa N., Hirayoshi K., Nagata K. (1994). Alternative 5′ splice site selection induced by heat shock. Mol. Cell. Biol..

[18] Fujikake N., Nagai Y., Popiel H.A., Kano H., Yamaguchi M., Toda T. (2005). Alternative splicing regulates the transcriptional activity of Drosophila heat shock transcription factor in response to heat/cold stress. FEBS Lett..

[19] Yang Y., Zhang C., Zhu D., He H., Wei Z., Yuan Q., Li X., Gao X., Zhang B., Gao H. (2022). Identifying candidate genes and patterns of heat-stress response in rice using a genome-wide association study and transcriptome analyses. Crop J..

[20] Sun J., Liu Z., Quan J., Li L., Zhao G., Lu J. (2022). RNA-seq Analysis Reveals Alternative Splicing Under Heat Stress in Rainbow Trout (Oncorhynchus mykiss). Mar. Biotechnol..

[21] Huang S., Dou J., Li Z., Hu L., Yu Y., Wang Y. (2022). Analysis of Genomic Alternative Splicing Patterns in Rat under Heat Stress Based on RNA-Seq Data. Genes.

[22] Pinto S., Hoffmann G., Ammon C., Amon B., Heuwieser W., Halachmi I., Banhazi T., Amon T. (2019). Influence of Barn Climate, Body Postures and Milk Yield on the Respiration Rate of Dairy Cows. Ann. Anim. Sci..

[23] Sammad A., Umer S., Shi R., Zhu H., Zhao X., Wang Y. (2020). Dairy cow reproduction under the influence of heat stress. J. Anim. Physiol. Anim. Nutr..

[24] Burfeind O., Suthar V.S., Heuwieser W. (2012). Effect of heat stress on body temperature in healthy early postpartum dairy cows. Theriogenology.

[25] Godyń D., Nowicki J., Herbut P. (2019). Effects of Environmental Enrichment on Pig Welfare—A Review. Animals.

[26] Dikmen S., Cole J.B., Null D.J., Hansen P.J. (2013). Genome-wide association mapping for identification of quantitative trait loci for rectal temperature during heat stress in Holstein cattle. PLoS ONE.

[27] Brito L.F., Oliveira H.R., McConn B.R., Schinckel A.P., Arrazola A., Marchant-Forde J.N., Johnson J.S. (2020). Large-Scale Phenotyping of Livestock Welfare in Commercial Production Systems: A New Frontier in Animal Breeding. Front. Genet..

[28] Mader T.L., Davis M.S., Brown-Brandl T. (2006). Environmental factors influencing heat stress in feedlot cattle1,2. Anim. Sci. J..

[29] Gaughan J.B., Mader T.L., Holt S.M., Sullivan M.L., Hahn G.L. (2010). Assessing the heat tolerance of 17 beef cattle genotypes. Int. J. Biometeorol..

[30] Gorniak T., Meyer U., Südekum K.-H., Dänicke S. (2014). Impact of mild heat stress on dry matter intake, milk yield and milk composition in mid-lactation Holstein dairy cows in a temperate climate. Arch. Anim. Nutr..

[31] West J.W. (2003). Effects of heat-stress on production in dairy cattle. J. Dairy Sci..

[32] Kim W.-S., Peng D.-Q., Jo Y.-H., Nejad J.G., Lee H.-G. (2021). Responses of beef calves to long-term heat stress exposure by evaluating growth performance, physiological, blood and behavioral parameters. J. Therm. Biol..

[33] Xiong Y., Yi H., Wu Q., Jiang Z., Wang L. (2020). Effects of acute heat stress on intestinal microbiota in grow-finishing pigs, and associations with feed intake and serum profile. J. Appl. Microbiol..

[34] McMorris T. (2016). Developing the catecholamines hypothesis for the acute exercise-cognition interaction in humans: Lessons from animal studies. Physiol. Behav..

[35] Guo K., Cao H., Zhu Y., Wang T., Hu G., Kornmatitsuk B., Luo J. (2018). Improving effects of dietary rumen protected gamma-aminobutyric acid additive on apparent nutrient digestibility, growth performance and health status in heat-stressed beef cattle. Anim. Sci. J..

[36] Habashy W.S., Milfort M.C., Rekaya R., Aggrey S.E. (2019). Cellular antioxidant enzyme activity and biomarkers for oxidative stress are affected by heat stress. Int. J. Biometeorol..

[37] Fang H., Kang L., Abbas Z., Hu L., Chen Y., Tan X., Wang Y., Xu Q. (2021). Identification of key Genes and Pathways Associated with Thermal Stress in Peripheral Blood Mononuclear Cells of Holstein Dairy Cattle. Front. Genet..

[38] Eisenhardt S.U., Thiele J.R., Bannasch H., Stark G.B., Peter K. (2009). C-reactive protein: How conformational changes influence inflammatory properties. Cell Cycle.

[39] Sonna L.A., Fujita J., Gaffin S.L., Lilly C.M. (2002). Invited Review: Effects of heat and cold stress on mammalian gene expression. J. Appl. Physiol..

[40] Sammad A., Luo H., Hu L., Zhu H., Wang Y. (2022). Transcriptome Reveals Granulosa Cells Coping through Redox, Inflammatory and Metabolic Mechanisms under Acute Heat Stress. Cells.

[41] Tan S., Wang W., Tian C., Niu D., Zhou T., Jin Y., Yang Y., Gao D., Dunham R., Liu Z. (2019). Heat stress induced alternative splicing in catfish as determined by transcriptome analysis. Comp. Biochem. Physiol. Part D Genom. Proteom..

[42] Bond U. (1988). Heat shock but not other stress inducers leads to the disruption of a sub-set of snRNPs and inhibition of in vitro splicing in HeLa cells. EMBO J..

[43] Black D.L. (2003). Mechanisms of Alternative Pre-messenger RNA Splicing. Annu. Rev. Biochem..

[44] Jiang J., Liu X., Liu C., Liu G., Li S., Wang L. (2017). Integrating Omics and Alternative Splicing Reveals Insights into Grape Response to High Temperature. Plant Physiol..

[45] Hao Y., Feng Y., Yang P., Cui Y., Liu J., Yang C., Gu X. (2016). Transcriptome analysis reveals that constant heat stress modifies the metabolism and structure of the porcine longissimus dorsi skeletal muscle. Mol. Genet. Genom..

[46] Liu S., Yue T., Ahmad M.J., Hu X., Zhang X., Deng T., Hu Y., He C., Zhou Y., Yang L. (2020). Transcriptome Analysis Reveals Potential Regulatory Genes Related to Heat Tolerance in Holstein Dairy Cattle. Genes.

[47] Keil L.C., Summy-Long J., Severs W.B. (1975). Release of Vasopressin by Angiotensin II1. Endocrinology.

[48] Mulrow P.J. (1999). Angiotensin II and aldosterone regulation. Regul. Pept..

[49] Shaji S., Sejian V., Bagath M., Manjunathareddy G.B., Kurien E.K., Varma G., Bhatta R. (2017). Summer season related heat and nutritional stresses on the adaptive capability of goats based on blood biochemical response and hepatic HSP70 gene expression. Biol. Rhy. Res..

[50] Tognacca R.S., Servi L., Hernando C.E., Saura-Sanchez M., Yanovsky M.J., Petrillo E., Botto J.F. (2019). Alternative Splicing Regulation During Light-Induced Germination of Arabidopsis thaliana Seeds. Front. Plant Sci..

[51] Carlson S.M., Soulette C.M., Yang Z., Elias J.E., Brooks A.N., Gozani O. (2017). RBM25 is a global splicing factor promoting inclusion of alternatively spliced exons and is itself regulated by lysine mono-methylation. J. Biol. Chem..

[52] Cheng C., Wang Z., Yuan B., Li X. (2017). RBM25 Mediates Abiotic Responses in Plants. Front. Plant Sci..

[53] Zhou A., Ou A.C., Cho A., Benz E.J., Huang S.C. (2008). Novel splicing factor RBM25 modulates Bcl-x pre-mRNA 5′ splice site selection. Mol. Cell Biol..

[54] Ouyang Y., Xia K., Yang X., Zhang S., Wang L., Ren S., Zhou H., Liu Y., Tang F. (2021). Alternative splicing acts as an independent prognosticator in ovarian carcinoma. Sci. Rep..

[55] Daniels N.J., Hershberger C.E., Gu X., Schueger C., DiPasquale W.M., Brick J., Saunthararajah Y., Maciejewski J.P., Padgett R.A. (2021). Functional analyses of human LUC7-like proteins involved in splicing regulation and myeloid neoplasms. Cell Rep..

[56] McDowell R.E., Hooven N.W., Camoens J.K. (1976). Effect of Climate on Performance of Holsteins in First Lactation. J. Dairy Sci..

[57] Luo H., Brito L.F., Li X., Su G., Dou J., Xu W., Yan X., Zhang H., Guo G., Liu L. (2021). Genetic parameters for rectal temperature, respiration rate, and drooling score in Holstein cattle and their relationships with various fertility, production, body conformation, and health traits. J. Dairy Sci..

[58] Rio D.C., Ares M., Hannon G.J., Nilsen T.W. (2010). Purification of RNA using TRIzol (TRI reagent). Cold Spring Harb. Protoc..

[59] Chen S., Zhou Y., Chen Y., Gu J. (2018). fastp: An ultra-fast all-in-one FASTQ preprocessor. Bioinformatics.

[60] Dobin A., Davis C.A., Schlesinger F., Drenkow J., Zaleski C., Jha S., Batut P., Chaisson M., Gingeras T.R. (2013). STAR: Ultrafast universal RNA-seq aligner. Bioinformatics.

[61] DeLuca D.S., Levin J.Z., Sivachenko A., Fennell T., Nazaire M.-D., Williams C., Reich M., Winckler W., Getz G. (2012). RNA-SeQC: RNA-seq metrics for quality control and process optimization. Bioinformatics.

[62] Robinson M.D., McCarthy D.J., Smyth G.K. (2010). edgeR: A Bioconductor package for differential expression analysis of digital gene expression data. Bioinformatics.

[63] Shen S., Park J.W., Lu Z.X., Lin L., Henry M.D., Wu Y.N., Zhou Q., Xing Y. (2014). rMATS: Robust and flexible detection of differential alternative splicing from replicate RNA-Seq data. Proc. Natl. Acad. Sci. USA.

[64] Li Y.I., Knowles D.A., Humphrey J., Barbeira A.N., Dickinson S.P., Im H.K., Pritchard J.K. (2018). Annotation-free quantification of RNA splicing using LeafCutter. Nat. Genet..

